# Measuring palliative care integration in Malawi through service provision, access, and training indicators: the Waterloo Coalition Initiative

**DOI:** 10.1186/s12904-023-01331-0

**Published:** 2024-01-16

**Authors:** Fatia Kiyange, Mackuline Atieno, Emmanuel B. K. Luyirika, Zipporah Ali, Helena Musau, Lameck Thambo, John Y. Rhee, Eve Namisango, William E. Rosa

**Affiliations:** 1 African Palliative Care Association, Kampala, Uganda; 2 Kenya Hospice and Palliative Care Association, Nairobi, Kenya; 3 Palliative Care Association of Malawi, Lilongwe, Malawi; 4 Dana-Farber Cancer Institute, Boston, MA USA; 5 Department of Psychiatry and Behavioral Sciences, Memorial Sloan Kettering Cancer Center, 633 Third Avenue, 4th Fl, New York, NY 10016 USA

**Keywords:** Palliative care, Primary palliative care, Morphine, Pain relief, Malawi, LMIC, Serious illness

## Abstract

**Background:**

Fewer than 1 in 20 people on the African continent in need of palliative care receive it. Malawi is a low-income country in sub-Saharan Africa that has yet to achieve advanced palliative care integration accompanied by unrestricted access to pain and symptom relieving palliative medicines. This paper studied the impact of Malawi’s Waterloo Coalition Initiative (WCI) – a local project promoting palliative care integration through service development, staff training, and increased service access.

**Methods:**

Interdisciplinary health professionals at 13 hospitals in southern Malawi were provided robust palliative care training over a 10-month period. We used a cross-sectional evaluation to measure palliative care integration based on 11 consensus-based indicators over a one-year period.

**Results:**

92% of hospitals made significant progress in all 11 indicators. Specifically, there was a 69% increase in the number of dedicated palliative care rooms/clinics, a total of 253 staff trained across all hospitals (a 220% increase in the region), substantive increases in the number of patients receiving or assessed for palliative care, and the number of hospitals that maintained access to morphine or other opioid analgesics while increasing the proportion of referrals to hospice or other palliative care programs.

**Conclusion:**

Palliative care is a component of universal health coverage and Sustainable Development Goal 3. The WCI has made tremendous strides in establishing and integrating palliative care services in Malawi with notable progress across 11 project indicators, demonstrating that increased palliative care access is possible in severely resource-constrained settings through sustained models of partnership at the local level.

**Supplementary Information:**

The online version contains supplementary material available at 10.1186/s12904-023-01331-0.

## Introduction

Despite much progress, global palliative care access inequities for people living in low- and middle-income countries (LMICs) are a worsening moral travesty requiring urgent and sustained clinical, health systems, and policy responses [1–5]. More than 61 million people worldwide are estimated to experience 6 billion days of serious health-related suffering that could be relieved with equitable access to palliative care [1, 6], achievable through country-specific actions [7–11], as well as local and global advocacy endeavors [12–14]. Yet nearly 90% of the global palliative care need is unmet and most of the unrelieved burden exists among people with life-limiting illness and injury in LMICs [1, 6]. The COVID-19 emergency unveiled pre-existing inequities and brought attention to the need for palliative care inclusion and increased availability of essential palliative care medicines in pandemic treaties and responses [15–19].

Africa accounts for about 20% of the global palliative care need among adults—mostly due to HIV/AIDS—and nearly 52% among children, largely as a result of progressive non-malignant conditions [6]. Although palliative care is a fundamental component of universal health coverage [20] and the right to the highest standards of mental and physical health [21], fewer than 1 in 20 people who need palliative care on the African continent receive it [6, 22]. Malawi is a low-income nation in sub-Saharan Africa with urgent need for increased palliative care infrastructure [23] (Table 1). Despite prevalent barriers to palliative care access (e.g., trained workforce, opioid phobia), Malawi’s palliative care development has accelerated in the past decade. In 2020, Malawi was categorized as a Group 4b country (i.e., palliative care at advanced stage of integration per *The Global Atlas of Palliative Care*) [6].

**Table 1:** Malawi: relevant background and data

Malawi is a landlocked country located in southeastern Africa that is bordered by Mozambique, Zambia and Tanzania. Malawi has a population 19,889,742 people, has a life expectancy of 62 years and has poverty count ratio of USD 2.15 a day of a population, and is ranked as the sixth poorest country in the world [23]. There is a high burden of disease, for example of the prevalence of HIV is 7.7 among adults, and over 990 000 people were living with HIV 2021 an incidence of 1.13 per 1000 population [24]. Malawi also faces a high burden of non-communicable diseases and an increase in their risk factors. For example, the estimated rates for heart disease, diabetes and hypertension are 9%, 6% and 16.7%, respectively [25]. The prevalence of obesity and smoking are also estimated to be 18.5% and 21.7% [26]. The health care services in Malawi are provided by three major players, the public, the private not for profit and the private for profit [27]. The prices for health services are subsidized in the private not for profit sector, and free in the public sector. In the private sector there are three major layers of service delivery, primary, secondary, and tertiary levels [28]. To achieve universal health coverage, the Ministry of Health Malawi supports palliative care service integration into existing services at all the three levels. That said, catastrophic out of pocket expenditures remain a problem for palliative care patients in Malawi. For example, research conducted in patients with advanced cancer demonstrates a high prevalence of catastrophic and out of pocket expenditure [29].	

The Waterloo Coalition Initiative (WCI) project aimed to integrate palliative care throughout public health systems in Malawi, thus improving access to palliative care services. The program was implemented by training health personnel, providing mentorship by palliative care organizations (such as Palliative Care Association of Malawi [30]), creating robust referral systems within and between hospitals, as well as between hospitals and lower levels of service delivery such as hospices and home-based palliative care services, and engaging in palliative care advocacy at a local level. The World Health Assembly recommended a comprehensive, integrated approach to palliative care service provision throughout the continuum of serious illness [20]. Thus, WCI serves as an exemplar at the local level, demonstrating a contextually appropriate innovation to achieve such an integrated approach. Using a diverse set of indicators to track palliative care integration through service development, staff training, and access, this paper reports on the major outcomes of the WCI in Malawi.

## Methods

The Palliative Care Support Trust, based at Queen Elizabeth’s Hospital in Blantyre, Malawi, was awarded funds by the African Palliative Care Association (APCA) [31] to develop and implement a STEP-UP program, led by the children’s palliative care service (Umodzi), as a way of expanding palliative care to the 13 hospitals in the southern region of Malawi, primarily by training health professionals. The training was led by palliative care providers from the Palliative Care Association of Malawi and APCA and delivered over a period of ten months. Training included increasing awareness of palliative care among the wider staff at each of the hospitals through continuing education; sensitization of local community health care professionals on availability of palliative care services; development of hospital-level services to optimize patient and family referral; support and training of hospices to serve as mentors to in-hospital palliative care units; and the development of hospital palliative care guidelines, including assessment forms, referral guidelines, morphine constitution and prescription guidelines, data collection forms, and provision of support. The training was based on a five-day introduction to palliative care course accredited by the Ministry of Health and the Palliative Care Association of Malawi. Clinical placements were part of the introductory training, although not all those who were trained were they were able to get clinical placements for on job training. Those trained were mainly health professionals (i.e., physicians, nurses, clinical officers/medical assistants) and allied health professionals (i.e., pharmacist technicians, laboratory technicians, physiotherapists, occupational therapists, dental therapists, surveillance assistants and orthopedic clinicians). Allied health professionals and clinicians were trained on a three-day pain management and use of opioids course. Administrators, such as accountants and human resource personnel, and community home-based care trainers, were also given a one-day orientation training. Supervision and mentorship were provided to effectively integrate palliative care services within hospital services; and palliative care resource centers were strengthened within the hospitals.

### Design

This was a cross-sectional census evaluation of 13 hospitals’ palliative care integration in the southern region of Malawi selected for participation by WCI. Hospitals were required to have established palliative care activity and a contact person. These inclusion criteria were intended to encourage progress and accountability to resolve challenges and difficulties in a timely manner across sites. Following a consensus meeting of palliative care providers in Malawi and APCA, a detailed framework was finalized on eleven indicators used to measure the project’s success in meeting objectives (Supplement 1).

### Data collection

Data were collected using a quantitative tool which captured all key indicators and narrative comments for the health service providers and senior managers to enhance further understanding of the data for each indicator (Supplement 2). The data collected included availability of opioids for medical use, number of patients with palliative care needs seen and referred, availability of protocols and guidelines, number of sensitization meetings held, and number of health professionals trained. The support included monitoring gaps in service delivering, and addressing them, such gaps included things like on job training, addressing stock outs for essential controlled medicines, ensuring the guidelines and protocols are in place and that sensitization to create awareness happens. These indicators were determined at the planning stages of the project with input from Monitoring and Evaluation teams at the participating hospitals and the criteria for selection was indicators that are essential for palliative care integration. Data collection and tracking process and tools to measure these outputs and outcomes were designed by the Monitoring and Evaluation team. The data were collected by a consultant who was trained and oriented on the tool prior to starting the assignment. The consultant was chosen based on his long-term experience in palliative care and understanding of the health systems in Malawi.

Data were collected from 13 hospitals in Malawi. Two of the hospitals were Health Center Hospitals (Balaka Hospital and Blantyre DHO/Mpemba Health Center), and eleven were District Level Hospitals (Chikhwawa Hospital, Chiradzulu Hospital, Machinga Hospital, Mangochi Hospital, Mwanza Hospital, Nneno Hospital, Nsanje Hospital, Phalombe DHO, Thyolo Hospital, Zomba DHO, and Mulanje Hospital).

We collected end of project data on the 11 agreed-upon indicators where the program was implemented (Supplement 1). Data collected focused on a three-month period (September – November 2012) for indicators 5, 6, 7, 8, 10; a cross-sectional snapshot in December 2012 for indicators 1,2, and 11 and over a one-year period at baseline (September 2010 – October 2011) and endpoint (January 2012 – December 2012) for indicators 3, 4 and 9.

As the study was part of a program evaluation process, administrative clearance to implement the programme was sought from the relevant authorities. Ethics and IRB approval was unnecessary since the project did not meet the definition of human subjects research per national research standards [32]. The protocol for the evaluation was shared with the Palliative Care Association of Malawi, which was involved in implementing the project.

## Results

Significant progress was made across all 11 indicators in 12 of the 13 hospitals (Table 2). Of note, there was one hospital (Blantyre DHO) with a significant decline in services due to organizational withdrawal of support during the three-month evaluation period. Indicator specific data is presented below.

**Table 2:** Malawi cross-sectional snapshot of performance indicators

Indicators^a^		Time	
		**Baseline**	**Endpoint**
1	Number of hospitals/health centers with established palliative care services	1	12
2	Number of hospitals/health centers with protocols and guidelines to include PC	1	13
3	Number of sensitization meetings held^b^	-	54
4	Number and type of health professionals trained in palliative care^c^	79	253
5	Number of patients receiving morphine	77	463
6	Number of patients receiving palliative care	72	1,463
7	Number of patients receiving pain relief	95	1,460
8	Number of patients being assessed for palliative care	938	1,482
9	Consistent supply of morphine and other palliative care medicines (based on one year period)	2	12
10	Number of patients currently being referred to other PC units	4	104
11	Number of hospitals/ service sites with patient discharge plans	10	12

^a^Indicators 1, 2, 3, 4, and 11 were obtained at a single-point in time, baseline at October 2011 and Endpoint on December 2012. Indicator 9 is based on a full-year recall. Indicators 5, 6, 7, 8, 9, and 10 are based on collection over a three-month baseline from July to September 2011 and three month endpoint of September to November 2012

^b^No baseline data

^c^Data collected over one year period

### Indicator 1: Number of hospitals with established palliative care services

At end of project, 12 of 13 health facilities had established palliative care services—a 92% increase in the number of hospitals with established palliative care services (Table 3). There was also progress in regard to the availability of a dedicated room/clinic for providing palliative care services. At endpoint, 12 of 13 hospitals had rooms/clinics, a 69% increase from baseline, with only 3 hospitals (Blantyre DHO, Thyolo Hospital, Zomba DHO) dedicating space to palliative care.

**Table 3:** Health system leve l indicator changes: from baseline to endpoint

Name of Health Facility	Has established palliative care services		Has stand-alone palliative care guidelines		Has palliative care integrated into the care guidelines/ protocols	
	**Baseline**	**Endpoint**	**Baseline**	**Endpoint**	**Baseline**	**Endpoint**
Balaka Hospital	No	Yes	No	No	No	Yes
Blantyre DHO (Mpemba Health Center)^a^	Yes	No	No	No	No	Yes
Chikhwawa Hospital	No	Yes	No	Yes	No	Yes
Chiradzulu Hospital	No	Yes	No	No	No	Yes
Machinga Hospital	No	Yes	No	No	No	Yes
Mangochi Hospital	No	Yes	No	No	No	Yes
Mulanje Hospital	No	Yes	No	Yes	No	Yes
Mwanza Hospital	No	Yes	No	Yes	No	Yes
Nneno Hospital	No	Yes	No	No	Yes	Yes
Nsanje Hospital	No	Yes	No	Yes	No	Yes
Phalombe DHO^a^	No	Yes	No	No	No	Yes
Thyolo Hospital	No	Yes	No	No	No	Yes
Zomba DHO^a^	No	Yes	No	Yes	No	Yes

^a^*DHO* District hospital

### Indicator 2: Number of hospitals with clinical protocols and guidelines that include palliative care

At endpoint, five hospitals/health centers had stand-alone palliative care guidelines and operating procedures, and 100% of hospitals had integrated palliative care into existing guidelines and/or protocols (Table 3). At baseline, only one hospital (Nneno Hospital) had palliative care integrated into existing guidelines and none of the hospitals had stand-alone guidelines or procedures.

### Indicator 3: Number of sensitization meetings held

No baseline data was collected for indicator 3. At endpoint, data showed that 12 of the 13 hospitals conducted some sensitization meetings on palliative care involving both management and technical teams (Blantyre DHO did not hold any meetings). A total of 54 sensitization meetings were held across all hospitals, ranging from one meeting at Machinga to eleven at Mulanje.

### Indicator 4: Number of staff members trained in palliative care

After training 12 of 13 hospitals/health centers, indicators showed an increase in the number of staff that had been trained in palliative care since baseline but with varying numbers. A total of 253 staff were trained across the 13 hospitals/health centers compared to only 79 trained staff at baseline, representing a 220% increase throughout the region. All hospitals had multidisciplinary palliative team members at endpoint with number of staff ranging from 6 at Nneno (excluding Blantyre, which had 3 at endpoint) to 66 at Phalombe. About (44%) of those trained were either clinical officers or nurses who are now leading provision of palliative care services at the hospitals/health centers.

### Indicator 5: Number of patients receiving morphine

**Fig. 1:**
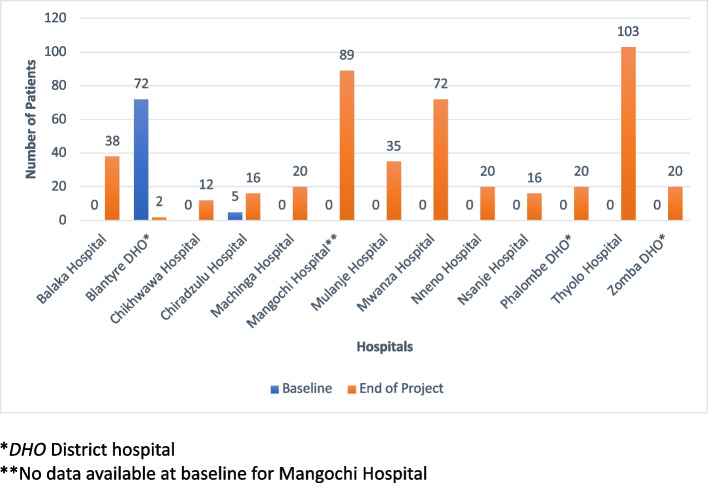
Number of patients receiving morphine at baseline and endpoint

At endpoint, 463 palliative care patients received morphine (mainly oral liquid and morphine sulphate tablets) at twelve hospitals compared to the baseline of 77 patients at two hospitals (Blantyre DHO and Chiradzulu Hospital). Forty (9%) of these patients were children receiving morphine at five hospitals (Balaka, Mangochi, Mwanza, Mulanje and Zomba DHO), indicating an improvement from baseline, where no pediatric patients were receiving morphine (Fig. 1).

### Indicator 6: Number of patients receiving palliative care

At baseline, only Blatyre DHO hospital was providing palliative care, with 72 patients receiving palliative care at the time. However, by endpoint, all the other twelve hospitals were providing palliative care, with the number of patients receiving palliative care having increased from 0 to 1,463, with numbers ranging from 20 patients (Nneno Hospital) to 568 (Mangochi Hospital) patients (excluding Blantyre DHO) (Table 4). Additionally, seven hospitals (Blantyre, Chikhwana, Chiradzulu, Mwanza, Nneno, Thyolo, and Zomba) provided nutritional support services, and an eighth (Mangochi) to patients on antiretroviral medications. All hospitals offered some level of psychological support and counseling. And nine hospitals provided spiritual support (not provided at Blantyre, Chiradzulu, Mwanza, and Zomba).

**Table 4:** Number of patients that received or were assessed for palliative care

**Hospital**	**Number of Patients Assessed Palliative Care**		**Number of Patients that Received Palliative Care**	
	**Baseline**	**Endpoint**	**Baseline**	**Endpoint**
Balaka Hospital	16	88	0	82
Blantyre DHO (Mpemba Health Center)^a^	72	13	72	0
Chikhwawa Hospital	40	49	0	49
Chiradzulu Hospital	23	43	0	52
Machinga Hospital	16	50	0	50
Mangochi Hospital	0	577	0	568
Mulanje Hospital	44	341	0	341
Mwanza Hospital	15	79	0	79
Nneno Hospital	0	20	0	20
Nsanje Hospital	20	21	0	21
Phalombe DHO^a^	20	34	0	34
Thyolo Hospital	7	124	0	124
Zomba DHO^a^	665	43	0	43
**Total**	**938**	**1482**	**72**	**1463**

^a^*DHO* District hospital

### Indicator 7: Number of patients receiving pain relief

A total of 1,460 palliative care patients received pain relief from across 12 hospitals and health centers compared to 95 patients from 2 hospitals/health centers at baseline. 125 (9%) were pediatric patients (increase from a baseline of 8 patients). Only three hospitals/health centers (i.e. Blantyre DHO, Nsanje Hospital, Thyolo Hospital) did not provide pain relief services to pediatric patients. Mangochi and Mulanje hospitals registered the highest numbers of patients accessing pain relief. These have enough staff on the palliative care teams and have established strong palliative care programs.

### Indicator 8: Number of people assessed for palliative care

The total number of people assessed for palliative care grew from a baseline of 938 to an endpoint of 1,482 patients (Table 4). All 13 hospitals are assessing patients for palliative care, compared to 11 (Mangochi and Nneno did not report having assessed any patients at baseline) at baseline. For all the hospitals/health centres, patients are routinely assessed for palliative care needs as part of the daily or weekly palliative care clinics. Mangochi runs regular palliative care clinic days while Nneno hospital is using various models of service delivery including home based palliative care. All hospitals/health centres except Zomba and Blantyre recorded an increase in the number of patients assessed for palliative care.

### Indicator 9: Consistent supply of morphine and other palliative care medicines

All hospitals except Blantyre DHO had strong opioids available most of the time. In addition, adjuvants and symptom controlling medications were available most of the time at all the 13 hospitals/health centers. However, weak opioids were only available most of the time in 5 of the 13 hospitals (Table 5).

**Table 5:** Changes in availability of strong opioids across the thirteen health facilities

Health Facility	Morphine Injectable	Morphine oral liquid	Morphine sulphate tablets	Pethidine	Methadone
Balaka Hospital	No	Yes^a^	Yes^c^	Yes^a^	No
Blantyre DHO^e^	No	No	No	No	No
Chikhwawa Hospital	No	Yes^a^	Yes^b^	Yes^a^	No
Chiradzulu Hospital	No	Yes^b^	Yes^a^	Yes^a^	No
Machinga Hospital	No	Yes^a^	Yes^c^	Yes^c^	No
Mangochi Hospital	No	Yes^a^	Yes^d^	Yes	Yes^d^
Mulanje Hospital	No	Yes^a^	Yes^b^	Yes^a^	No
Mwanza Hospital	No	Yes^a^	Yes^c^	Yes	No
Nneno Hospital	Yes^a^	Yes^b^	Yes^c^	Yes	No
Nsanje Hospital	No	Yes^a^	Yes^d^	Yes^a^	No
Phalombe DHO^e^	No	Yes	Yes^b^	Yes	No
Thyolo Hospital	No	Yes^a^	Yes^b^	Yes	No
Zomba DHO^e^	No	Yes	No	No	No

^a^Out-of-stock 5% of the time

^b^Out-of-stock 25% of the time

^c^Out-of-stock 50% of the time

^d^Out-of-stock 80% of the time

^e^*DHO* District hospital

At endpoint, a total of 12 hospitals compared to only 2 hospitals at baseline (i.e. Blantyre DHO and Chiradzulu Hospital) had a consistent supply of strong opioids, especially oral liquid morphine and morphine sulphate tablets, which were available most of time. Oral Liquid morphine and morphine sulphate tablets are not available at only one hospital (i.e. Blantyre DHO), which was a significant decline from baseline for this hospital; it was one of the two facilities that had strong opioids and other essential medications at baseline.

A total of nine hospitals and health centers had oral liquid morphine for 95% of the time, while 2 hospitals and facilities had it for 75% of the time. Nine hospitals had codeine, of which five had it in stock 50% of the time or more. Only two hospitals (Chiradzulu and Thyolo) had tramadol, although it was frequently out of stock. All hospitals reported having adjuvants and other symptom-control medications in stock, with the majority rarely or never being out of stock.

### Indicator 10: Number of patients currently being referred to other palliative care sites

A total of 153 referrals were made overall with 104 (68%) of these palliative care referrals, up from 4 (1%) of the total referrals at baseline (298). These came from 12 of the hospitals/health centers with Zomba DHO making referrals, but not having recorded them. The increase in the number of palliative care referrals is consistent with the increase in the number of hospitals (12) making referrals by end of the project compared to only 2 hospitals at baseline (i.e. Chikhwawa Hospital and Thyolo Hospital). Most of the referrals were made to hospitals/facilities with palliative care services as well as hospice programs. Table 6 presents data on palliative care referrals by hospital and type of referral and a comparison between baseline and end of project.

**Table 6:** Changes in palliative care referrals

**Health Facility**	**Referred to Hospice**		**Referred to Other Palliative Care Program**		**Total Palliative Care Referrals**	
	**Baseline**	**End-point**	**Baseline**	**End-point**	**Baseline totals**	**Endpoint totals**
Balaka Hospital	0	4	0	7	0	11
Blantyre DHO^b^	0	13	0	0	0	13
Chikhwawa Hospital	0	3	1	3	1	6
Chiradzulu Hospital	0	3	0	0	0	3
Machinga Hospital	0	2	0	0	0	2
Mangochi Hospital	0	0	0	3	0	3
Mulanje Hospital	0	5	0	20	0	25
Mwanza Hospital	0	8	0	25	0	33
Nneno Hospital	0	1	0	1	0	2
Nsanje Hospital	0	0	0	1	0	1
Phalombe DHO^b^	0	1	0	1	0	2
Thyolo Hospital	0	0	3	3	3	3
Zomba DHO^a, b^	0	-	0	-	0	-
**Total**	**0**	**40**	**4**	**64**	**4**	**104**

^a^Endpoint data unable to be collected

^b^*DHO* District hospital

### Indicator 11: Number of hospitals with a patient discharge plan

Twelve (92%) hospitals/health centers had written discharge plans, up from 10 hospitals (77%) at baseline. All the 13 hospitals always gave a referral form to the patient. Eight hospitals always notified the organization where a referral is being made. Table 7 below shows the details of patient discharge plans.

**Table 7:** Changes in referral and discharge processes

Health Facility	Hospital has written discharge plan for patient	Patient given a referral form	Hospital notifies the organization where patient is referred
Balaka Hospital	Yes	Yes	Yes
Blantyre DHO^b^	Yes	Yes	No
Chikhwawa Hospital	Yes	Yes	Yes
Chiradzulu Hospital	Yes	Yes	No
Machinga Hospital	Yes	Yes	Yes
Mangochi Hospital	Yes	Yes	Yes
Mulanje Hospital	Yes	Yes	No
Mwanza Hospital	Yes	Yes	Yes
Nneno Hospital	Yes	Yes	Yes
Nsanje Hospital	Yes	Yes	- ^a^
Phalombe DHO^b^	No	Yes	No
Thyolo Hospital	Yes	Yes	Yes
Zomba DHO^b^	Yes	Yes	Yes

^a^The one patient referred was within the hospital so no prior communication

^b^*DHO* District hospital

## Discussion

This study aimed to evaluate outcomes of the WCI – a 10-month project that integrated palliative care throughout 13 hospitals in the southern region of Malawi—thus enhancing availability of services across eleven indicators, primarily through the training of health professionals. Our assessment demonstrated a 92% increase in the number of hospitals or health centers with reliable palliative care services in Malawi, a low-income country in sub-Saharan Africa [6]. Through sustained partnerships at the institutional, local, and regional levels, the WCI shows that increased availability of palliative care is possible through a staunch commitment to enhanced referral systems, mentorship between experts and generalists, and strategic implementation of evidence-based guidelines. The WCI provides a model for palliative care integration in other LMICs that can be adapted and contextualized based on need and available physical, financial, and human resources.

One major global public health injustice that prevents universal health coverage worldwide is the global palliative care and pain relief divide, cited by international experts and disproportionately impacting the poorest populations [1]. The recent World Health Organization (WHO) report on global variations in safe access to morphine for medical use found that, in a global survey of multi-sector stakeholders (*n*= 356 responses) across all six WHO regions, low-income country respondents (50%) and lower-middle income respondents (18%) reported at least 8 in 10 people did not receive morphine or other strong opioids in times of medical need due to inconsistent availability [33]. Twelve of the 13 hospitals participating in the WCI showed that strong opioids, including oral liquid morphine and morphine sulphate tablets, as well as non-opioid adjuvants and other symptom controlling medications, were available most of the time throughout the study period. In short, our palliative care integration project nudged hospitals in the southern region of Malawi closer to ensuring the crucial and most basic medicines required for effective and evidence-based palliative care provision (e.g., for treatment of moderate to severe pain and terminal dyspnea). Where palliative care was not included as a stand-alone guideline but was integrated into existing guidelines and/or protocols, it was usually included in the District Implementation Plan (DIP) of the hospital and/or drug budget. This is often a preference for countries which may not have standalone funds to support vertical components of care and they prefer to integrate the palliative care into existing funded policies to make the implementation practical.

There are a number of clinical implications of the WCI. All hospitals noted a need for ongoing sensitization of senior hospital management and other health care workers as well as for further education, including specialized training for nurses leading palliative care units. Health workforce capacity building is a core component of developing robust palliative care delivery pathways – not only to cultivate pain and symptom management competencies, but also to advocate for the improved sustained provision of humane and crucial services to relieve suffering. The components of service provision beyond the management of the physical domain need further strengthening, as many of the hospitals referred patients to external resources for psychosocial and spiritual care. These findings suggest a need for clinicians and hospitals to build active and mutually beneficial partnerships with external service providers and organizations who can collaborate to deliver holistic, person-centered care. While referral to community-based mental health, spiritual care, or palliative specialists may be necessary for complex symptom burdens or refractory suffering, every health professional can be trained in basic palliative care skills (e.g., generalist care to attend to holistic care needs). Considering this approach, nurses, social workers, chaplains, and physicians, can all participate in psychological, social, and spiritual care needs assessments and screenings to improve care and referral planning, mitigate disruption during care transitions, and enhance overall team communication to meet patient and carer needs.

Future research should investigate palliative care integration approaches that build on the findings of the WCI, ensuring a broad capture of baseline data for longitudinal measurement purposes, expanding sites over time in accordance with resources to leverage impact across health delivery settings, and considering implementation science designs to maximize benefit for populations who may likely lack needed access for health-related suffering in the context of serious illness. The WCI emphasized the need to integrate palliative care in keeping with institutional norms and values while focusing on interdisciplinary and multi-sector partnerships between clinicians, organizations, health systems, and experts. Community-based participatory research approaches can help investigative teams to remain population focused and to meet the needs of the population being served while meeting the goals of local partners [34–36]. As researchers continue to identify better methods to measuring palliative care need and assessing palliative care integration, it will be essential to ensure that workforce capacity building remains at the center of study design and evaluation.

To our knowledge, this is the first study to report on a regional palliative care integration initiative that demonstrates increased access of services and necessary medicines in Malawi. Although some findings have measured palliative care development through country-level indicators, hospital-level indicators often give more information as to the variability, distribution, and kinds of services available for provision within a specific country. Such data is crucial in the Global South where resources are severely constrained and understanding of palliative care frequently remains in nascent stages [1, 6].

Our findings should be considered in the context of the following limitations. The WCI was launched in 2012 and data presented here are more than a decade old. However, these results support resource-constrained settings to identify and adapt key strategies that can be implemented over time to drive palliative care integration. As of 2017, mapping of palliative care development and integration into public facilities showed that Malawi was one of two African nations to achieve an advanced stage of integration (stage 4b), alongside Eswatini (then known as Swaziland) [6, 37]. Other developments since the WCI include Malawi’s launching of a standalone policy for palliative care, integration of palliative care into medical and nursing curricula, and a supply chain mechanism for opioid distribution [38]. Other limitations include our inability to obtain baseline data for hospital and health center referrals, opioid availability, or proportion of time that opioids were available. Yet measures of service availability and referrals remains substantially important; the explicit availability of different types of opioids across regional hospitals is vital to describing the current state of development of palliative care in the country. Some indicators were measured at different endpoints, complicating cross-sectional comparisons at a specific point in time. However, the data provides a novel empirical contribution to the literature regarding hospital-level indicators of palliative care access. Resource and funding constraints limited the team to a singular data collection consultant, resulting in the inability to independent cross-checks. Given that the data were mostly quantitative in measure, the risk for bias or error in data collection was minimal, especially since the consultant was external and not a hospital employee.

## Conclusion

Sustainable Development Goal target 3.8 states that every person should have universal access to high-quality and affordable health services [39]—universal palliative care access is fundamental to achieving this vision [40]. The WCI in Malawi has made tremendous strides in establishing and integrating palliative care services in the country with notable progress across eleven project indicators, including health workforce capacity development, improved access to opioids and other symptom management medications, and more robust referral pathways. More importantly, the WCI demonstrates that increased palliative care access is possible in severely resource-constrained settings through sustained models of partnership that prioritize patient wellbeing and quality health service provision.

### Supplementary Information

**Additional file 1: Supplement 1.** Description of project indicators.

**Additional file 2: Supplement 2.** Indicator collection tool.

## Data Availability

All data or analyzed during this study are included in the published article and its supplementary information files.
