# Supplementary material for: Measuring palliative care integration in Malawi through service provision, access, and training indicators: the Waterloo Coalition Initiative
Source: BMC Palliat Care. 2024 Jan 16;23:17. doi: 10.1186/s12904-023-01331-0 (PMC10790398; doi:10.1186/s12904-023-01331-0)
Supplement: Supplementary file 1 — Additional file 1: Supplement 1. Description of project indicators. [file 12904_2023_1331_MOESM1_ESM.docx]

**Supplement 1. Description of Project Indicators.**

| **Indicators** | | **Indicator description** |
| --- | --- | --- |
| 1 | Number of hospitals/health centers with established palliative care services | This indicator was used to determine whether a hospital had an established palliative care service using the following criteria: 1) Palliative care provision on-site within the hospital (not through referral to a local hospice); 2) Presence of a dedicated room/clinic for palliative care with coordinating staff; 3) Consistent supply of morphine and other palliative care drugs including liquid morphine; 4) Availability of trained staff; 5) Routine administration of palliative care assessments |
| 2 | Number of hospitals/health centers with protocols and guidelines to include PC | This indicator measures the inclusion of palliative care in the hospital clinical guidelines as evidenced by details regarding 1) The components or aspects of palliative care; 2) The type of guidelines/protocol where included; 3) The existence of standalone palliative care guidelines/protocols is also referred to by this indicator. |
| 3 | Number of sensitization meetings held | This indicator measures awareness raising and/or advocacy sessions/meetings on palliative care and highlights the numbers of people sensitized. |
| 4 | Number and type of health professionals trained in palliative care | This indicator measures number of hospital staff members trained in palliative care. |
| 5 | Number of patients receiving morphine. | This indicator measures the inclusion of morphine on the National Essential Medicines List or a requirement by hospital policy for morphine to be made available to patients who need it. It also measures the extent of “stock-outs” experienced in a one-year period, based on various formulations of morphine (injectable morphine, oral liquid morphine, or morphine sulphate tablets). It specifically measures amounts of liquid morphine used for palliative care, not post-operatively. |
| 6 | Number of patients receiving palliative care. | In measuring this indicator, the definition of “receiving palliative care” included patient’s access to assessment, pain relief, and the management of other physical symptoms, psychosocial and spiritual support across the continuum of care. |
| 7 | Number of patients receiving pain relief | This includes use of strong and weak opioids in relieving patient’s pain. |
| 8 | Number of patients being assessed for palliative care | This indicator assessed the total number of people that were assessed for palliative care over this time period. |
| 9 | Consistent supply of morphine and other palliative care medicines (based on one year period) | Morphine and other palliative care drugs needed to be available 75% of the time, when recording the data over a one-year period, to indicate a consistent supply. |
| 10 | Number of patients currently being referred to other PC units | Data were obtained for all patients with palliative care needs who left the hospital in order to track referrals. Patient referrals were categorized as follows: 1) discharged home (discharged from the hospital without being referred to any other service provider); 2) referred to a home-based care support program, which is not a palliative care program; 3) referred to a home-based palliative care program; 4) referred to a palliative care in-patient program. |
| 11 | Number of hospitals/ service sites with patient discharge plans. | Data collected was based on the total number of hospitals/health centers with individual patient discharge plans as well the content of the discharge or referral form, if available. |
