# Supplementary material for: Measuring palliative care integration in Malawi through service provision, access, and training indicators: the Waterloo Coalition Initiative
Source: BMC Palliat Care. 2024 Jan 16;23:17. doi: 10.1186/s12904-023-01331-0 (PMC10790398; doi:10.1186/s12904-023-01331-0)
Supplement: Supplementary file 2 — Additional file 2: Supplement 2. Indicator collection tool. [file 12904_2023_1331_MOESM2_ESM.docx]

| **Data Collection Tool: Waterloo Project** | |
| --- | --- |
| **Issued Version: May 2012** | |
| **Instructions** | |
| **Please read these instructions before attempting to complete use this spreadsheet** | |
| **Purpose of Data Collection Tool** | |
| In July 2011, the Waterloo Coalition approved two grants to support the integration of palliative care into public health systems in Malawi and Kenya by increasing the scope of and access to palliative care in 11 government hospitals in Kenya, and in 13 in Malawi. The African Palliative Care Association is responsible for monitoring and evaluating these projects to ensure that they achieve the targeted outcomes. | |
| The purpose of this spreadsheet is to collect project information from each of the 24 hospitals on Palliative Care service provision. This information collected will be used to track the success of the project by measuring progress through data collection during project implementation. | |
| **Process and timings** | |
| The data collection process is being supported by two separate roles, APCA on one hand, and KEHPCA and PCST on the other hand. KEHPCA and PCST will be responsible for sending out this data collection template and receiving it back again. The APCA lead is responsible for consolidating the information, reporting on it, and answering any technical queries on spreadsheet completion. The named leads are as follows: | |
| ***Support:***  If you require technical support in the completion of this template, or have queries on methodology or definitions, please contact the APCA lead and the in-country leads on those emails and phone numbers. | |
| **Overview of the Data Collection Tool** | |
| **Sheets** | |
| This data collection tool contains 2 sheets: 1. Site Information: The purpose of this is to ensure understanding of the operational context.  2. Indicator Information: The purpose of this is to collect information on the indicators for the Waterloo project | |
| **Protection** | |
| The tool has been protected for ease of use. All input cells should be completed. | |
| **Period** | |
| The data collection is to be done on quarterly basis. For a description of the baseline period for each question, please consult the final column in each sheet. | |
| **Instruction Details** | |
| High level instructions are given below this text. For more detailed instructions on completion of each question, please hover your mouse over the question number which appears in column A of each sheet, and a clear definition of the information being collected will appear. | |
| **Site Information Sheet** | |
| This sheet collects information on the hospital. This is used for contextual purposes, not for monitoring and evaluation. It is anticipated that the information collected in this sheet should all be readily available in existing documents / databases. If it is not readily available, please contact the in-country lead to discuss. | |
| The following information is collected: 1. Hospital contact information 2. Information on hospital and district 3. Information on patient number and profile and beds 4. Staffing information | |
| **Indicator Information Sheet** | |
| **Palliative Care:** The term "Palliative Care", as used throughout the indicator information sheet means care given to patients with life-limiting illness aimed at improving their quality of life, by addressing their "total pain": physical, psychological, social and spiritual. | |
| **Section 1: Availability of Palliative Care Service** | The purpose of this section is to gather baseline information on Indicator 1 of this project: number of hospitals with Palliative Care Services. Information is gathered on whether the hospital has an established Palliative Care Service, the nature of this service, the aspects of Palliative Care offered and numbers of people trained. |
| **Section 2: Palliative Care Guidelines** | The purpose of this section is to gather information on Indicator 2 of this project: number of protocols developed to include Palliative Care. Information is gathered on the nature of protocols (stand-alone/incorporated) |
| **Section 3: Patients Receiving Palliative Care** | The purpose of this section is to gather information on Indicators 5 - 8 of this project: number of patients assessed for and receiving PC, and receiving morphine, or other pain relief. We recognise that it may be difficult for some hospitals to count information on number of patients assessed for PC, unless they have a dedicated team, or an appropriate space on a patient assessment form. We have therefore asked for detail on the methodology used to gather this. |
| **Section 4: Availability of Essential Medicines** | The purpose of this section is to gather more information on indicators 5 and 7, number of patients receiving pain relief and morphine. The section also measures the consistency of supply of morphine and other Palliative Care drugs. |
| **Section 5: Patient Referral Process** | The purpose of this section is to gather more information on indicators 9 and 10, number of patients being referred to other PC units, and number of hospitals with patient discharge plans. The number of patients counted should only be those patients with Palliative Care needs. Because the quality of the referral process is extremely important, information is also gathered on the documentation and process surrounding referrals. |

|  | **SITE INFORMATION** |  |  |
| --- | --- | --- | --- |
|  | **Version: May 2012** |  | ***Period*** |
|  |  |  |  |
|  | Name: Project lead within hospital |  |  |
|  | Name: Person who completed tool |  |  |
|  | Contact number |  |  |
|  | Contact email |  |  |
|  |  |  |  |
| ***Hospital information*** | | | |
| 1 | Country |  |  |
| 2 | Hospital |  |  |
| 3 | Level |  |  |
|  |  |  |  |
| ***Catchment Area*** | | | |
| 5 | Name of County |  |  |
| 6 a. | Catchment population |  | Latest data available as of Dec 2012 |
| 6 b. | Period/Source of population figure |  |  |
|  |  |  |  |
| ***Beds and Patients*** | | | |
| 7 a. | Total Beds: Adult |  | Snapshot as of December 2012 |
| 7 b. | Total Beds: Paediatric |  | Snapshot as of December 2012 |
| 8 a. | Total inpatients: Adult |  | In the last 3 months |
| 8 b. | Total inpatients: Paediatric |  | In the last 3 months |
| 9 a. | Total outpatients: Adult |  | In the last 3 months |
| 9 b. | Total outpatients: Paediatric |  | In the last 3 months |
|  | **Total inpatients and outpatients** |  |  |
|  |  |  |  |
| 10 a. | Total patients: Male |  | In the last 3 months |
| 10 b. | Total patients: Female |  | In the last 3 months |
|  |  |  |  |
|  |  |  |  |
| ***11. Top 5 reasons for admission in the hospital*** | | | |
| 11a |  | | In the last 3 months |
| 11b |  | | In the last 3 months |
| 11c |  | | In the last 3 months |
| 11d |  | | In the last 3 months |
| 11e |  | | In the last 3 months |
|  |  |  |  |
| ***12. Total staff employed*** |  |  |  |
| ***Medical and Nursing*** | | | |
| 12a | Doctors |  | Snapshot as of December 2012 |
| 12b | Nurses |  | Snapshot as of December 2012 |
| 12c | Clinical Officers |  | Snapshot as of December 2012 |
|  |  |  |  |
| ***Professions allied to health*** | |  | Snapshot as of December 2012 |
| 12d | Social Workers |  | Snapshot as of December 2012 |
| 12e | Physiotherapists |  | Snapshot as of December 2012 |
| 12f | Occupational Therapists |  | Snapshot as of December 2012 |
| 12g | Counsellors |  | Snapshot as of December 2012 |
| 12h | Pharmacy Staff |  | Snapshot as of December 2012 |
| 12i | Nutitionists |  | Snapshot as of December 2012 |
| 12j | Dental |  | Snapshot as of December 2012 |
| 12k | Other Allied Health Professionals |  | Snapshot as of December 2012 |
|  |  | |  |
|  |  |  |  |
| ***Other Staff*** | |  | Snapshot as of December 2012 |
| 12l | Administrators / Management |  | Snapshot as of December 2012 |
| 12m | Non clinical/admin support staff |  | Snapshot as of December 2012 |
| 12n | Other |  | Snapshot as of December 2012 |
|  |  | |  |
|  |  |  |  |
|  | **Total Staff** |  | Snapshot as of December 2012 |

|  | **INDICATOR INFORMATION** |  |  |  |  |  |  |
| --- | --- | --- | --- | --- | --- | --- | --- |
|  | **Version: May 2012** |  |  |  |  |  |  |
|  | **SECTION 1: AVAILABILITY OF PALLIATIVE CARE SERVICE** |  |  |  |  |  | ***Period*** |
|  |  |  |  |  |  |  |  |
| 1 a.i | Does the hospital have an established PC service? |  |  |  |  |  | Snapshot - December 2012 |
| 1 a.ii | Does the hospital have a dedicated Palliative Care Team / Individual? |  |  |  |  |  | Snapshot - December 2012 |
| 1 a.iii | How many people are in this team? |  |  |  |  |  | Snapshot - December 2012 |
| 1 a.iv | Have any other staff (in addition to the team above) been trained in PC? |  |  |  |  |  |  |
|  |  |  |  |  |  |  |  |
| ***1b Please give numbers trained in PC, and detail of training by cadre*** | | ***total*** | ***details*** | | |  |  |
| 1 b.i | Doctors |  |  | | |  | Since October 2011 |
| 1 b.ii | Nurses / Clinical Officers |  |  | | |  | Since October 2011 |
| 1 b.iii | Allied Health Professional |  |  | | |  | Since October 2011 |
| 1 b.iv | Managers / Administrators |  |  | | |  | Since October 2011 |
| 1 b.v | Other (please specify) |  |  | | |  | Since October 2011 |
|  |  | **0** |  |  |  |  | Since October 2011 |
|  |  |  |  |  |  |  |  |
| ***1c Services offered to patients at the hospital*** | |  | |  |  |  |  |
| 1 c.i | Pain Relief |  | |  | |  | In the last 3 months |
| 1 c.ii | Symptom Control |  | |  | |  | In the last 3 months |
| 1 c.iii | Adherence to medications |  | |  | |  | In the last 3 months |
| 1 c.vi | Nutritional Support |  | |  | |  | In the last 3 months |
| 1 c.v | Counselling / Psychological Support |  | |  | |  | In the last 3 months |
| 1 c.vi | Spiritual Support |  | |  | |  | In the last 3 months |
| 1 c.vii | Social Support |  | |  | |  | In the last 3 months |
|  |  |  |  |  |  |  |  |
|  | **SECTION 2: PALLIATIVE CARE GUIDELINES** |  |  |  |  |  |  |
|  |  | **Yes/ No** |  | **Details** |  |  |  |
| 2 b.i | Does the hospital have stand-alone Palliative Care guidelines? |  |  | | |  | Snapshot - December 2012 |
| 2 b.ii | Does the hospital have PC integrated into hospital guidelines/protocols? |  |  | | |  | Snapshot - December 2012 |
|  |  |  |  |  |  |  |  |
|  | **SECTION 3: PATIENTS RECEIVING PALLIATIVE CARE** |  |  |  |  |  |  |
|  |  | **Yes/No** |  | **Details** |  |  |  |
| 3 a | Are patients routinely assessed for palliative care needs in this hospital? |  |  | | |  | Snapshot - December 2012 |
|  |  |  |  |  |  |  |  |
| **3 b** | Give the number of patients ***assessed for*** Palliative Care |  |  |  |  |  |  |
| 3 b.i | Paediatric |  |  | | |  | In the last 3 months |
| 3 b.ii | Adults |  |  |  |  |  | In the last 3 months |
| **3 b.iii** | **Total patients assessed for Palliative Care** | **0** |  |  |  |  |  |
|  |  |  |  |  |  |  |  |
| **3 c** | Give the number of patients ***receiving*** palliative care |  |  |  |  |  |  |
| 3 c.i | Paediatric |  |  | | |  | In the last 3 months |
| 3 c.ii | Adults |  |  |  |  |  | In the last 3 months |
| **3 c.iii** | **Total patients receiving Palliative Care** | **0** |  |  |  |  |  |
|  |  |  |  |  |  |  |  |
| **3 d** | Give the number of patients ***receiving pain relief*** |  |  |  |  |  |  |
| 3 d.i | Paediatric |  |  | | |  | In the last 3 months |
| 3 d.ii | Adults |  |  |  |  |  | In the last 3 months |
| **3 d.v** | **Total patients receiving pain relief** | **0** |  |  |  |  | In the last 3 months |
|  |  |  |  |  |  |  |  |
| **3 e.** | Give the number of patients ***receiving morphine*** |  |  |  |  |  |  |
| 3 e.i | Paediatric |  |  | | |  | In the last 3 months |
| 3 e.ii | Adults |  |  |  |  |  | In the last 3 months |
| **3 e.iii** | **Total patients receiving morphine** | **0** |  |  |  |  | In the last 3 months |
|  |  |  |  |  |  |  |  |
|  | **SECTION 4: AVAILABILITY OF ESSENTIAL MEDICATIONS** |  |  |  |  |  |  |
|  | **Availability of morphine and other palliative care medicines** |  |  |  |  |  |  |
| ***4 a. Is there a consistent supply of morphine and other palliative care medicines?*** | | ***Yes/ No*** | | ***Stock-outs*** | |  |  |
| 4 a.i | Morphine |  | |  | |  | In the last 1 year |
| 4 a.ii | Other PC medicines |  | |  | |  | In the last 1 year |
|  |  |  |  |  |  |  |  |
| ***4 b. What type of morphine are you currently using for patients with palliative care needs?*** | |  |  |  |  |  |  |
| 4 b.i | Morphine - injectable |  | |  | |  | In the last 1 year |
| 4 b.ii | Morphine - oral liquid |  | |  | |  | In the last 1 year |
| 4 b.iii | Morphine sulphate tablets |  | |  | |  | In the last 1 year |
|  |  |  |  |  |  |  |  |
| **4 c** | **Do you experience stock-outs of morphine and other PC medicines?** |  | |  | |  | In the last 1 year |
|  |  |  |  |  |  |  |  |
| **4 d** | **If no, which medicines suffer from stock-outs? Specify** |  | | | |  |  |
|  |  |  |  |  |  |  |  |
|  | **SECTION 5: PALLIATIVE CARE PATIENT REFERRAL PROCESS** |  |  |  |  |  |  |
| ***5 a. Number of patients being referred to hospice or other support programmes*** | | ***total*** | ***details*** |  |  |  |  |
| 5 a.i | Discharged home |  |  | | |  | In the last 3 months |
| 5 a.ii | Referred to Hospice |  |  | | |  | In the last 3 months |
| 5 a.iii | Referred other palliative care support programme |  |  | | |  | In the last 3 months |
| 5 a.iv | **Total referred to PC programme** | **0** |  |  |  |  |  |
|  |  |  |  |  |  |  |  |
| 5 a.v | If other support programme, specify under details |  | | | |  |  |
|  |  |  |  |  |  |  |  |
| ***5 b. Does the hospital have an individual discharge plan*** | |  |  |  |  |  |  |
| 5 b.i | Does the hospital have a written discharge plan for each patient? |  |  |  |  |  | Snapshot - December 2012 |
| 5 b.ii | Is the patient always given a referral form? |  | |  |  |  | Snapshot - December 2012 |
| 5 b.iii | Does the hospital notify the organisation/ hospital where the ppatient is referred? |  | |  |  |  | Snapshot - December 2012 |
|  |  |  |  |  |  |  |  |
| 5 d. | Is there any further information on palliative care you would like to provide to us? |  | | | |  |  |
